# Supplementary material for: Sleep does not influence schema-facilitated motor memory consolidation
Source: PLoS One. 2023 Jan 19;18(1):e0280591. doi: 10.1371/journal.pone.0280591 (PMC9851548; doi:10.1371/journal.pone.0280591)
Supplement: S1 Fig — (PDF) [file pone.0280591.s001.pdf]

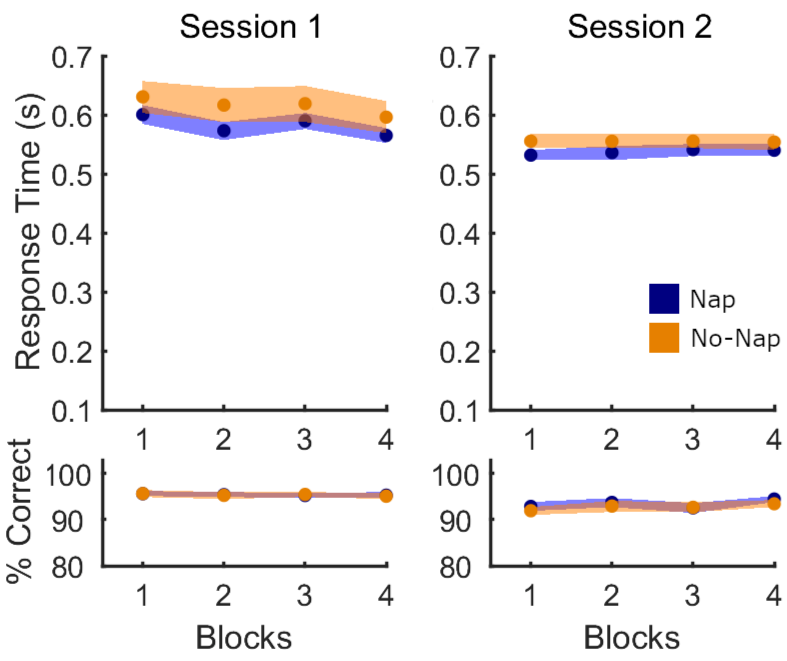

S1 Fig: Performance on the pseudo-random SRTT in Experiment 1 (N=25 in each of the two groups). Mean response time (in seconds; top panel) and % correct transitions per block of task (bottom) are depicted separately for Session 1 and Session 2. Output of the corresponding statistical analyses is provided in S2 Table.
