# Supplementary material for: Sleep does not influence schema-facilitated motor memory consolidation
Source: PLoS One. 2023 Jan 19;18(1):e0280591. doi: 10.1371/journal.pone.0280591 (PMC9851548; doi:10.1371/journal.pone.0280591)
Supplement: S2 Fig — (PDF) [file pone.0280591.s002.pdf]

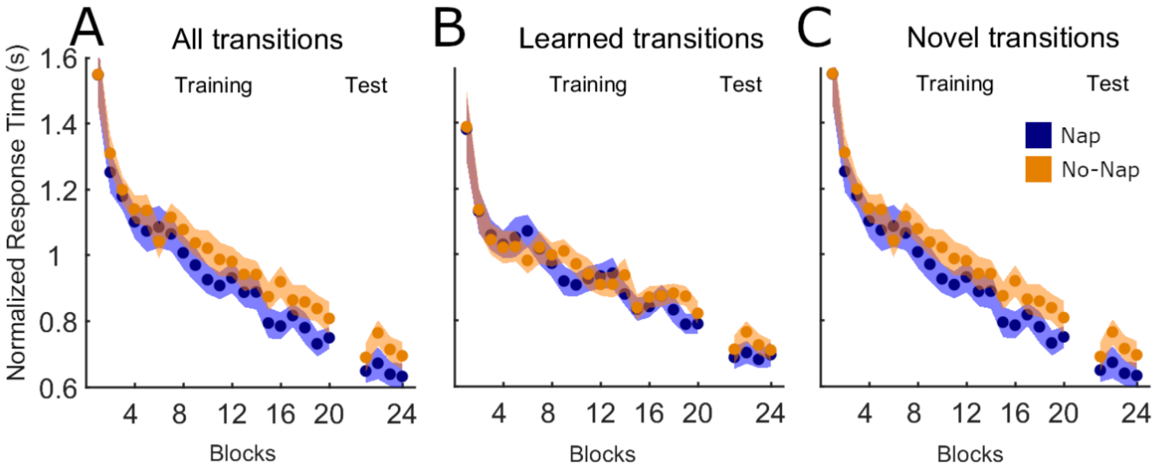

Figure S2: Normalized response time during Session 2 training and test runs for all transitions (**A**), learned transitions (**B**), and novel transitions (**C**) for the two groups in Experiment 1 (N=25 in each of the two groups). Shaded areas represent the SEM. Normalization was computed by dividing the response time per each block and participant by that individual's mean response time during the 4 blocks of Session 1 test. After normalization, the trend towards a significant group difference observed in the non-normalized data (see main text, Figures 2 and 3) disappeared, as evidenced by the lack of group effects or group x block interaction. Detailed statistics are as follows:

All transitions – training – main effect group: $F(1,48)=0.65$, $p=0.42$, $\eta^2=0.013$; block × group interaction: $F(3.7,176.4)=0.59$, $p=0.66$, $\eta^2=0.012$; test – main effect group: $F(1,46)=2.09$, $p=0.16$, $\eta^2=0.043$; block × group interaction: $F(2.5,112.7)=0.31$, $p=0.78$, $\eta^2=0.007$.

Learned transitions – training –main effect group: $F(1,48)=0.09$, $p=0.77$, $\eta^2=0.002$; block × group interaction: $F(4.2,203.2)=0.75$, $p=0.57$, $\eta^2=0.015$; test – main effect group: $F(1,46)=1.14$, $p=0.29$, $\eta^2=0.024$; block × group interaction: $F(2.2,101.0)=0.25$, $p=0.73$, $\eta^2=0.008$.

Novel transitions – training –main effect group: $F(1,48)=1.39$, $p=0.25$, $\eta^2=0.028$; block × group interaction: $F(4.0,191.2)=0.47$, $p=0.76$, $\eta^2=0.010$; test – main effect group: $F(1,46)=1.87$, $p=0.18$, $\eta^2=0.039$; block × group interaction: $F(2.7,126.0)=0.13$, $p=0.93$, $\eta^2=0.003$.
