# Supplementary material for: Sleep does not influence schema-facilitated motor memory consolidation
Source: PLoS One. 2023 Jan 19;18(1):e0280591. doi: 10.1371/journal.pone.0280591 (PMC9851548; doi:10.1371/journal.pone.0280591)
Supplement: S3 Fig — (PDF) [file pone.0280591.s003.pdf]

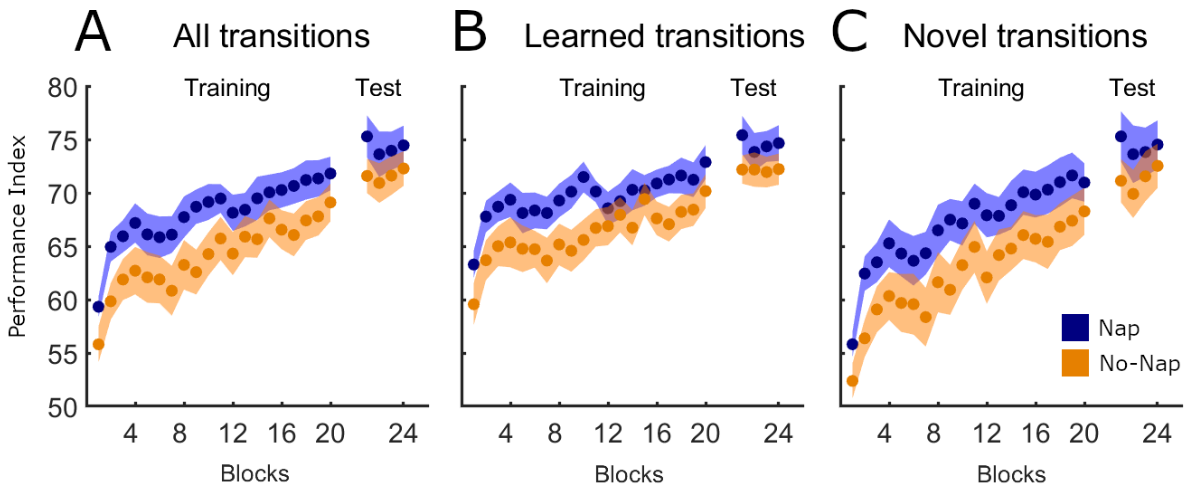

S3 Fig. Aggregate speed-accuracy measure performance index (PI) during session 2 of Experiment 1 (N=25 in each of the 2 groups) is depicted for all (**A**), learned (**B**) and novel transitions (**C**). A higher PI index corresponds to better performance. Corresponding statistical analyses can be found in S4 Table. Shaded areas represent the SEM.
