# Supplementary material for: Sleep does not influence schema-facilitated motor memory consolidation
Source: PLoS One. 2023 Jan 19;18(1):e0280591. doi: 10.1371/journal.pone.0280591 (PMC9851548; doi:10.1371/journal.pone.0280591)
Supplement: S4 Fig — (PDF) [file pone.0280591.s004.pdf]

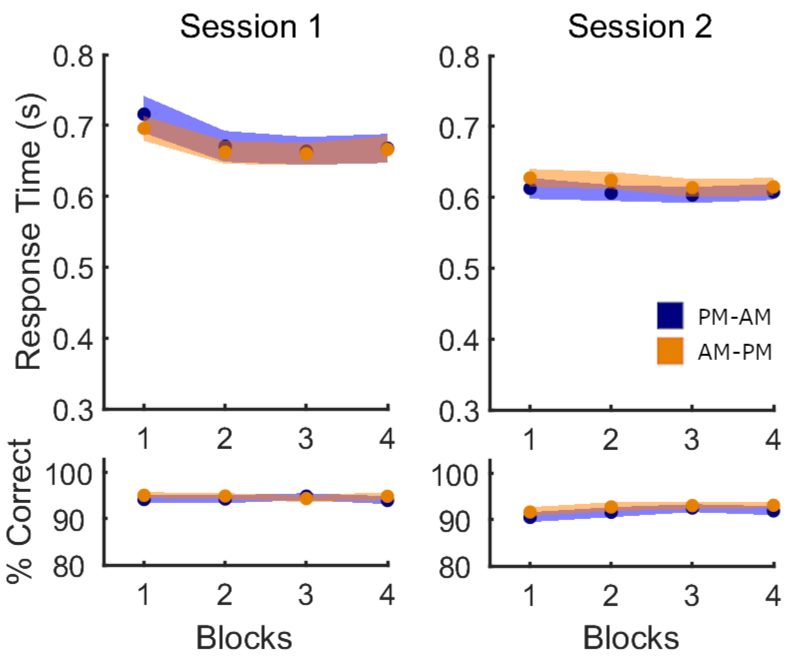

S4 Fig: Performance on the pseudo-random SRTT in Experiment 2 (AM-PM group: N=29; PM-AM group: N=28). Mean response time (in seconds) and % correct transitions per block of sequence task are depicted for the two sessions. Output of the corresponding statistical analyses is provided in S11 Table.
