# Supplementary material for: Sleep does not influence schema-facilitated motor memory consolidation
Source: PLoS One. 2023 Jan 19;18(1):e0280591. doi: 10.1371/journal.pone.0280591 (PMC9851548; doi:10.1371/journal.pone.0280591)
Supplement: S1 Table — (PDF) [file pone.0280591.s005.pdf]

S1 Table: Group differences in participant characteristics and assessments of sleep and vigilance for Experiment 1.

**A. Variable**	**t**	**p**	**Cohen's d**
Age	-1.29	0.20	-0.366
BAI score	0.90	0.38	0.253
BDI score	0.21	0.83	0.060
Handedness score	-0.26	0.80	-0.073
PSQI score	0.67	0.51	0.188
Daytime sleepiness score	-1.68	0.10	-0.475
Sleep duration, 3 nights prior to S2	-0.56	0.58	-0.159
SMS duration	-0.53	0.60	-0.150
SMS quality	0.20	0.84	0.058
**B. SSS**	**F**	**p**	**Partial η^2**
Session	0.47	0.50	0.010
Session x Group	0.83	0.37	0.017
Group	0.62	0.43	0.013
**C. PVT**	**F**	**p**	**Partial η^2**
Session	1.04	0.31	0.021
Session x Group	0.001	0.98	<0.001
Group	0.27	0.60	0.006

Output of statistical analyses assessing group differences (Nap vs. No Nap) in participant characteristics, sleep quality and quantity prior to the experimental sessions as well as subjective (Stanford Sleepiness Scale (SSS)[1]) and objective (Psychomotor Vigilance Task (PVT)[2]) assessments of vigilance. Means and SDs are provided in Table 1 of the main text. Variables in section **A** were assessed with independent samples t-tests (df = 48 for all). SSS (section **B**) and PVT (**C**) were assessed with 2 (Session) by 2 (Group) ANOVAs (df = 1,48 for all effects). No significant Group, Session or Group by Session effects were revealed. BAI = Beck's anxiety inventory [3]; BDI = Beck's depression inventory [4]; PSQI = Pittsburgh Sleep Quality Index [5], SMS = St. Mary's sleep questionnaire [6]; S2= session 2. N=25 in each of the two groups.

References

1. Hoddes E, Dement W, Zarcone V. The development and use of the Stanford sleepiness scale (SSS). *Psychophysiology*. 1972;9:150.
2. Dinges DF, Powell JW. Microcomputer analyses of performance on a portable, simple visual RT task during sustained operations. *Behav Res Methods, Instruments, Comput*. 1985;17(6):652-655. doi:10.3758/BF03200977
3. Beck AT, Epstein N, Brown G, Steer RA. An inventory for measuring clinical anxiety: Psychometric properties. *J Consult Clin Psychol*. 1988;56(6):893-897. doi:10.1037/0022-006X.56.6.893
4. Beck AT, Steer RA, Ball R, Ranieri WF. Comparison of Beck depression inventories -IA and -II in psychiatric outpatients. *J Pers Assess*. 1996;67(3):588-597. doi:10.1207/s15327752jpa6703_13
5. Buysse DJ, Reynolds CF, Monk TH, Berman SR, Kupfer DJ. The Pittsburgh sleep quality index: A new instrument for psychiatric practice and research. *Psychiatry Res*. 1989;28(2):193-213. doi:10.1016/0165-1781(89)90047-4
6. Ellis BW, Johns MW, Lancaster R, Raptopoulos P, Angelopoulos N, Priest RG. The St. Mary's Hospital sleep questionnaire: a study of reliability. *Sleep*. 1981;4(1):93-97. doi:10.1093/SLEEP/4.1.93
