# Supplementary material for: Sleep does not influence schema-facilitated motor memory consolidation
Source: PLoS One. 2023 Jan 19;18(1):e0280591. doi: 10.1371/journal.pone.0280591 (PMC9851548; doi:10.1371/journal.pone.0280591)
Supplement: S2 Table — (PDF) [file pone.0280591.s006.pdf]

S2 Table: Performance on the pseudo-random SRTT in Experiment 1.

Effect	df	F	p	Partial η^2
**A. Response Time (RT)**				
Session 1				
Block	2.5,121.9	11.59	<0.001*	0.195
Block x Group	2.5,121.9	0.59	0.60	0.012
Group	1,48	1.18	0.28	0.024
Session 2				
Block	3,144	0.45	0.72	0.009
Block x Group	3,144	0.61	0.61	0.012
Group	1,48	1.40	0.24	0.028
**B. Accuracy**				
Session 1				
Block	3,144	0.26	0.86	0.005
Block x Group	3,144	0.06	0.98	0.001
Group	1,48	0.002	0.97	<0.001
Session 2				
Block	3,144	3.11	0.03	0.061
Block x Group	3,144	0.44	0.73	0.009
Group	1,48	0.30	0.59	0.006
**C. Performance Index (PI)**				
Session 1				
Block	3,144	5.50	0.001*	0.103
Block x Group	3,144	0.56	0.64	0.012
Group	1,48	1.04	0.31	0.021
Session 2				
Block	3,144	1.88	0.14	0.038
Block x Group	3,144	0.80	0.50	0.016
Group	1,48	1.27	0.27	0.026

Output of statistical analyses assessing group differences in performance on the pseudo-random SRT task assessing general motor execution administered prior to and following the sequential SRT task in Sessions 1 and 2, respectively. Separate 4 (Block) by 2 (Group) ANOVAs were run per each variable (**A**: Response Time, RT; **B**: Accuracy; and **C**: Performance Index, PI) and each Session. The presence of a significant effect of block during Session 1 for RT and PI indicates a general improvement in performance, presumably due to task familiarization (see Supplementary Figure 1, Session 1). No main effect of group or block x group interaction were observed for any measure or in either session, demonstrating that general motor execution did not differ between experimental groups. Df = degrees of freedom. N=25 in each of the two groups.
