# Supplementary material for: Sleep does not influence schema-facilitated motor memory consolidation
Source: PLoS One. 2023 Jan 19;18(1):e0280591. doi: 10.1371/journal.pone.0280591 (PMC9851548; doi:10.1371/journal.pone.0280591)
Supplement: S3 Table — (PDF) [file pone.0280591.s007.pdf]

S3 Table: Performance on the generation task in Experiment 1.

Variable	Nap	No-Nap	t(48)	p	Cohen's d
Session 1					
% correct transitions	66.1 (34.8)	49.9 (35.2)	1.63	0.11	0.462
% correct ordinal positions	56.8 (36.6)	44.3 (37.5)	1.19	0.24	0.337
Session 2					
% correct transitions	82.3 (27.8)	68.7 (30.9)	1.63	0.11	0.462
% correct ordinal positions	72.3 (35.4)	57.3 (37.6)	1.45	0.15	0.411

Numbers in the Nap and No Nap columns represent the means, with standard deviation in parentheses. We observed no group differences in knowledge of the motor sequences learned in Session 1 and Session 2 of Experiment 1, as measured by % correct transitions and % correct ordinal positions self-generated by the participants. N=25 in each of the two groups.
