# Supplementary material for: Sleep does not influence schema-facilitated motor memory consolidation
Source: PLoS One. 2023 Jan 19;18(1):e0280591. doi: 10.1371/journal.pone.0280591 (PMC9851548; doi:10.1371/journal.pone.0280591)
Supplement: S4 Table — (PDF) [file pone.0280591.s008.pdf]

S4 Table: Output of statistical analyses performed on PI in Session 2 of Experiment 1.

Effect	df	F	p	Partial η²
A. All transitions				
Training				
Block	8.7,429.1	23.84	<0.001*	0.332
Block x Group	8.7,429.1	0.54	0.84	0.011
Group	1,48	3.04	0.09	0.060
Test				
Block	3,138	1.19	0.32	0.025
Block x Group	3,138	0.32	0.81	0.007
Group	1,46	1.36	0.25	0.029
B. Learned transitions				
Training				
Block	9.2,442.2	9.87	<0.001*	0.170
Block x Group	9.2,442.2	0.83	0.59	0.017
Group	1,48	3.07	0.09	0.060
Test				
Block	3,138	0.43	0.73	0.009
Block x Group	3,138	0.23	0.87	0.005
Group	1,46	1.56	0.22	0.033
**C. *Novel transitions***				
Training				
Block	9.7,463.5	26.09	<0.001*	0.352
Block x Group	9.7,463.5	0.40	0.94	0.008
Group	1,48	2.38	0.11	0.054
Test				
Block	3,138	1.55	0.21	0.033
Block x Group	3,138	0.62	0.61	0.013
Group	1,46	1.09	0.30	0.023
D. Transition type x Group				
Transition type	1,48	0.23	0.64	0.005
Transition type x Group	1,48	0.13	0.72	0.003
Group	1,48	1.34	0.25	0.027

Output of statistical analyses on Performance Index (PI) during Session 2 of Experiment 1. Block x Group ANOVAs were computed for the training and test runs for all (**A**), learned (**B**) and novel (**C**) transitions. Additionally, a 2 (Transition type) by 2 (Group) ANOVA was also computed for performance during the test phase (**D**). These analyses correspond to those performed on the primary variables RT and Accuracy (see Figures 2-3 and Table 3 in main text) and obtained largely the same results: a significant effect of block was present for all transitions as well as for both transition subtypes, but there was no significant effect of group or group x block interaction. Performance also did not differ between the two transition types (learned and novel) nor experimental groups. (N=25 and N=24 in each group for assessments of Training and Test runs, respectively).
