# Supplementary material for: Sleep does not influence schema-facilitated motor memory consolidation
Source: PLoS One. 2023 Jan 19;18(1):e0280591. doi: 10.1371/journal.pone.0280591 (PMC9851548; doi:10.1371/journal.pone.0280591)
Supplement: S5 Table — (PDF) [file pone.0280591.s009.pdf]

S5 Table: Average sleep characteristics for participants in the Nap group (Experiment 1), per each midline channel.

	Mean (Standard Deviation)
**A. Fz**	
Spindle density (n/min)	1.58 (1.30)
Spindle amplitude (μV)	41.42 (11.02)
Slow wave density (n/min)	4.19 (4.26)
Slow wave peak-to-peak amplitude (μV)	127.76 (18.36)
**B. Cz**	
Spindle density (n/min)	1.70 (1.29)
Spindle amplitude (μV)	43.45 (10.76)
Slow wave density (n/min)	3.85 (3.73)
Slow wave peak-to-peak amplitude (μV)	119.21 (19.23)
**C. Pz**	
Spindle density (n/min)	1.96 (1.30)
Spindle amplitude (μV)	37.86 (9.44)
Slow wave density (n/min)	3.38 (3.68)
Slow wave peak-to-peak amplitude (μV)	119.79 (18.84)

Sleep characteristics during the 90-minute nap opportunity for participants in the Nap group (Experiment 1) for channels Fz (A), Cz (B), and Pz (C). Spindle and slow wave characteristics were automatically detected in NREM sleep epochs using the YASA python package[1]. As three participants did not have any slow waves detected on channel Pz, N=22 for correlations with slow wave density and amplitude; N=25 for all other correlations. Further details are provided in the main text (Methods – Experiment 1).

References

1. Vallat R. raphaelvallat/yasa: v0.1.9. Zenodo. doi:doi:10.5281/ZENODO.3646596
