# Supplementary material for: Sleep does not influence schema-facilitated motor memory consolidation
Source: PLoS One. 2023 Jan 19;18(1):e0280591. doi: 10.1371/journal.pone.0280591 (PMC9851548; doi:10.1371/journal.pone.0280591)
Supplement: S6 Table — (PDF) [file pone.0280591.s010.pdf]

S6 Table: Correlations between sleep features (Nap group only) and performance in the sequential SRT task during Session 2 for Experiment 1.

	**All transitions**	**Learned transitions**	**Novel transitions**
**A. Performance Index**			
NREM duration	r=-0.12; p=0.73	r=-0.30; p=0.33	r=0.02; p=0.92
Spindle density	r=-0.05; p=0.81	r=0.10; p=0.63	r=-0.16; p=0.77
Spindle amplitude	r=-0.40; p=0.25	r=-0.22; p=0.36	r=-0.45; p=0.10
Slow wave density	r=-0.33; p=0.28	r=-0.27; p=0.33	r=-0.32; p=0.30
Slow wave amplitude	r=-0.20; p=0.55	r=-0.30; p=0.33	r=-0.10; p=0.78
**B. Response Time**			
NREM duration	r=0.06; p=0.78	r=0.10; p=0.80	r=0.03; p=0.88
Spindle density	r=0.08; p=0.78	r=-0.02; p=0.91	r=0.15; p=0.73
Spindle amplitude	r=0.36; p=0.38	r=0.35; p=0.40	r=0.34; p=0.33
Slow wave density	r=0.30; p=0.38	r=0.27; p=0.50	r=0.31; p=0.33
Slow wave amplitude	r=0.16; p=0.75	r=0.21; p=0.53	r=0.12; p=0.73
**C. Accuracy**			
NREM duration	r=0.04; p=0.99	r=-0.26; p=0.65	r=0.21; p=0.80
Spindle density	r=-0.02; p=0.99	r=0.02; p=0.92	r=-0.03; p=0.88
Spindle amplitude	r=-0.13; p=0.99	r=0.24; p=0.65	r=-0.30; p=0.75
Slow wave density	r=0.11; p=0.99	r=0.10; p=0.85	r=0.06; p=0.88
Slow wave amplitude	r=-0.004; p=0.99	r=-0.09; p=0.85	r=0.06; p=0.88

Correlations between sleep features averaged across channels Fz, Cz, and Pz, and sequential SRT performance in Session 2 (post-sleep), as measured by the Performance Index (**A**), Response Time (**B**) and Accuracy (**C**). For all three variables, performance was normalized by dividing the mean across the 20 training blocks of session 2 sequential SRTT by the average performance on the 4 blocks of pseudorandom SRTT completed during session 2. No significant correlations were observed. All reported values are adjusted for multiple comparisons (False Discovery Rate, FDR correction). Note that correlations between Performance Index and learned and novel transitions were part of our pre-registered analyses, whereas all others were considered exploratory. N=25 for all correlations.
