# Supplementary material for: Sleep does not influence schema-facilitated motor memory consolidation
Source: PLoS One. 2023 Jan 19;18(1):e0280591. doi: 10.1371/journal.pone.0280591 (PMC9851548; doi:10.1371/journal.pone.0280591)
Supplement: S9 Table — (PDF) [file pone.0280591.s013.pdf]

S9 Table: Correlations between sleep features (Nap group only) and performance in the sequential SRT task during Session 2 for Experiment 1, channel Pz.

	**All transitions**	**Learned transitions**	**Novel transitions**
**A. Performance Index**			
Spindle density	r=0.10; p=0.63	r=0.20; p=0.45	r=0.02; p=0.94
Spindle amplitude	r=-0.26; p=0.36	r=-0.10; p=0.62	r=-0.32; p=0.32
Slow wave density	r=-0.30; p=0.36	r=-0.23 p=0.45	r=-0.31; p=0.32
Slow wave amplitude	r=-0.25; p=0.36	r=-0.23; p=0.45	r=-0.22; p=0.44
**B. Response Time**			
Spindle density	r=-0.06; p=0.78	r=-0.12; p=0.57	r=-0.02; p=0.95
Spindle amplitude	r=0.21; p=0.47	r=0.23; p=0.55	r=0.19; p=0.48
Slow wave density	r=0.27; p=0.47	r=0.19; p=0.55	r=0.31; p=0.48
Slow wave amplitude	r=0.21; p=0.47	r=0.19; p=0.55	r=0.22; p=0.48
**C. Accuracy**			
Spindle density	r=0.03; p=0.95	r=0.06; p=0.97	r=-0.01; p=0.97
Spindle amplitude	r=-0.15; p=0.95	r=0.25; p=0.92	r=-0.33; p=0.44
Slow wave density	r=0.08; p=0.95	r=-0.01; p=0.97	r=0.09; p=0.97
Slow wave amplitude	r=-0.01; p=0.95	r=-0.06; p=0.97	r=0.02; p=0.97

Correlations between sleep features detected on channel Pz and sequential SRT performance in Session 2 (post-sleep), as measured by the Performance Index (**A**), Response Time (**B**) and Accuracy (**C**). For all three variables, performance was normalized by dividing the mean across the 20 training blocks of session 2 sequential SRTT by the average performance on the 4 blocks of pseudorandom SRTT completed during session 2. No significant correlations were observed. All reported values are adjusted for multiple comparisons (FDR correction). Note that these correlations were not part of our pre-registered analyses and are thus considered exploratory. As three participants did not have any slow waves detected on channel Pz, N=22 for correlations with slow wave density and amplitude; N=25 for correlations with spindle density and amplitude.
