# Supplementary material for: Sleep does not influence schema-facilitated motor memory consolidation
Source: PLoS One. 2023 Jan 19;18(1):e0280591. doi: 10.1371/journal.pone.0280591 (PMC9851548; doi:10.1371/journal.pone.0280591)
Supplement: S10 Table — (PDF) [file pone.0280591.s014.pdf]

S10 Table: Participant characteristics and assessments of sleep and vigilance for Experiment 2.

A. Variable	t	p	Cohen's d
Age	-0.84	0.41	-0.221
BAI score	0.94	0.35	0.250
BDI score	0.15	0.88	0.039
Handedness score	-0.04	0.97	-0.010
PSQI score	0.54	0.59	0.144
Daytime sleepiness score	0.04	0.97	0.009
Sleep duration, 3 nights prior to S2	1.76	0.08	0.471
SMS duration	1.50	0.14	0.397
SMS quality	1.03	0.31	0.273
**B. SSS**	**F**	**p**	**Partial η^2**
Session	2.06	0.16	0.036
Session x Group	0.54	0.47	0.010
Group	0.02	0.90	<0.001
**C. PVT**	**F**	**p**	**Partial η^2**
Session	4.80	0.03*	0.080
Session x Group	1.33	0.25	0.024
Group	0.29	0.59	0.005

Output of statistical analyses assessing group differences (AM-PM vs. PM-AM) in participant characteristics, sleep quality and quantity prior to the experimental sessions as well as subjective (Stanford Sleepiness Scale (SSS)[1]) and objective (Psychomotor Vigilance Task (PVT)[2]) assessments of vigilance. Means and SDs are provided in Table 1 of the main text. Variables in section **A** were assessed with independent samples t-tests (df = 55 for all). SSS (section **B**) and PVT (**C**) were assessed with 2 (Session) by 2 (Group) ANOVAs (df = 1,55 for all effects). BAI = Beck's anxiety inventory [3]; BDI = Beck's depression inventory [4]; PSQI = Pittsburgh Sleep Quality Index [5], SMS = St. Mary's sleep questionnaire [6]; S2= session 2. No significant Group, Session or Group by Session effects were revealed with the exception of a significant effect of Session for the PVT. This was driven by overall decreased response time in Session 2 (276 ± 44 ms, compared with 286 ± 55 ms in Session 1). Importantly, this decrease across sessions did not differ between groups, as indicated by the lack of a Session x Group interaction. AM-PM group: N=29; PM-AM group: N=27 for 'Sleep duration, 3 nights prior to S2', N=28 for all else.

References

1. Hoddes E, Dement W, Zarcone V. The development and use of the Stanford sleepiness scale (SSS). *Psychophysiology*. 1972;9:150.
2. Dinges DF, Powell JW. Microcomputer analyses of performance on a portable, simple visual RT task during sustained operations. *Behav Res Methods, Instruments, Comput*. 1985;17(6):652-655. doi:10.3758/BF03200977
3. Beck AT, Epstein N, Brown G, Steer RA. An inventory for measuring clinical anxiety: Psychometric properties. *J Consult Clin Psychol*. 1988;56(6):893-897. doi:10.1037/0022-006X.56.6.893
4. Beck AT, Steer RA, Ball R, Ranieri WF. Comparison of Beck depression inventories -IA and -II in psychiatric outpatients. *J Pers Assess*. 1996;67(3):588-597. doi:10.1207/s15327752jpa6703_13
5. Buysse DJ, Reynolds CF, Monk TH, Berman SR, Kupfer DJ. The Pittsburgh sleep quality index: A new instrument for psychiatric practice and research. *Psychiatry Res*. 1989;28(2):193-213. doi:10.1016/0165-1781(89)90047-4
6. Ellis BW, Johns MW, Lancaster R, Raptopoulos P, Angelopoulos N, Priest RG. The St. Mary's Hospital sleep questionnaire: a study of reliability. *Sleep*. 1981;4(1):93-97. doi:10.1093/SLEEP/4.1.93
