# Supplementary material for: Sleep does not influence schema-facilitated motor memory consolidation
Source: PLoS One. 2023 Jan 19;18(1):e0280591. doi: 10.1371/journal.pone.0280591 (PMC9851548; doi:10.1371/journal.pone.0280591)
Supplement: S11 Table — (PDF) [file pone.0280591.s015.pdf]

S11 Table: Performance on pseudo-random SRTT in Experiment 2.

**Effect**	**df**	**F**	**p**	**Partial η²**
**A. Response Time (RT)**				
Session 1				
Block	2.6,143.4	19.96	<0.001*	0.266
Block x Group	2.6,143.4	0.79	0.48	0.014
Group	1,55	0.10	0.76	0.002
Session 2				
Block	3,165	2.21	0.09	0.039
Block x Group	3,165	0.50	0.69	0.009
Group	1,55	0.59	0.45	0.011
**B. Accuracy**				
Session 1				
Block	2.8,153.9	0.05	0.98	0.001
Block x Group	2.8,153.9	0.57	0.63	0.010
Group	1,55	036	0.55	0.006
Session 2				
Block	3,165	3.38	<0.05*	0.058
Block x Group	3,165	0.16	0.93	0.003
Group	1,55	0.79	0.38	0.014
**C. Performance Index (PI)**				
Session 1				
Block	2.7,145.8	7,64	0.001*	0.103
Block x Group	2.7,145.8	0.57	0.64	0.012
Group	1,55	0.20	0.31	0.021
Session 2				
Block	3,165	4.73	0.003*	0.079
Block x Group	3,165	0.18	0.91	0.003
Group	1,55	0.02	0.88	<0.001

Output of statistical analyses assessing group differences in performance on the pseudo-random SRT task administered prior to and following the sequential SRT task in Sessions 1 and 2, respectively, reflecting general motor execution. Separate 4 (Block) by 2 (Group) ANOVAs were run per each variable (**A**: Response Time, RT; **B**: Accuracy) and each Session. The presence of a significant effect of Block during Session 1 for RT and PI indicates a general increase in performance, presumably due to task familiarization. The significant effect of *block* on accuracy in Session 2 was caused by a marginal decrease of an overall very high accuracy during the first block (average 91% accuracy, compared with 92% in the other three blocks. No main effect of group or block x group interaction were observed for any measure, in either session, demonstrating that general motor execution did not differ between experimental groups. Df = degrees of freedom. AM-PM group: N=29; PM-AM group: N=28.
