# Supplementary material for: Sleep does not influence schema-facilitated motor memory consolidation
Source: PLoS One. 2023 Jan 19;18(1):e0280591. doi: 10.1371/journal.pone.0280591 (PMC9851548; doi:10.1371/journal.pone.0280591)
Supplement: S12 Table — (PDF) [file pone.0280591.s016.pdf]

S12 Table: Performance on the generation task in Experiment 2.

Variable	AM-PM	PM-AM	t(55)	p	Cohen's d
Session 1					
% correct transitions	13.3 (24.8)	7.8 (11.7)	1.08	0.28	0.287
% correct ordinal positions	24.0 (23.1)	19.2 (12.3)	0.97	0.34	0.257
Session 2					
% correct transitions	43.5 (40.6)	52.5 (40.4)	-0.84	0.41	-0.223
% correct ordinal positions	48.3 (37.8)	59.4 (35.0)	-1.15	0.26	-0.304

Numbers in the AM-PM and PM-AM columns represent the means, with standard deviation in parentheses. We observed no group differences in knowledge of the motor sequences learned in Session 1 and Session 2 of Experiment 1, as measured by % correct transitions and % correct ordinal positions self-generated by the participants. AM-PM group: N=29; PM-AM group: N=28.
