# Supplementary material for: Sleep does not influence schema-facilitated motor memory consolidation
Source: PLoS One. 2023 Jan 19;18(1):e0280591. doi: 10.1371/journal.pone.0280591 (PMC9851548; doi:10.1371/journal.pone.0280591)
Supplement: Attachment — Submitted filename: Response to Reviewers.pdf [file pone.0280591.s017.pdf]

<u>ID number of the original manuscript</u>: PONE-D-22-16102

<u>Manuscript title</u>: "Sleep does not influence schema-facilitated motor memory consolidation"

We would like to thank the editor for the time devoted to our manuscript and for giving us the opportunity to submit a revised version of our paper to *PLOS ONE*. We also thank the reviewers for their constructive comments. We have addressed all of their points in the detailed responses below. All changes listed in this letter are highlighted in yellow in the revised version of the main text. [Note that changes to supplemental files are provided in this response document, but not tracked in the updated versions in order to adhere to *PLoS One* submission guidelines.] We believe that the manuscript has improved and thus hope that the editor and the reviewers will find our changes satisfactory.

<u>Editor:</u>

Comment 1: In my view, the submission does not meet the journal's requirements for the "minimal data set" availability. Although data was provided to recreate the graphs, data is not currently available to replicate the study findings. While providing data to produce graphs is described as an example on the data availability policy description (https://journals.plos.org/plosone/s/data-availability), this should be interpreted in the context of a multi-discipline journal with discipline specific practices in regard to analysis and sharing. For psychology, the standard practice would be to make subject level available that provided the inputs to the analysis, though ideal best practice may be to include trial level data. Therefore I would ask for subject level data to be made available to allow these analyses to be replicated or alternative analyses performed.

<u>Author response:</u> The data file attached to the project indeed included subject level data that served as the input to the statistical analyses outlined in the manuscript. Accordingly, interested individuals could replicate the study findings with the data file given. However, we agree with the editor that the best practice is to include trial level data. Our intention was to make the raw data publicly available upon publication but we are happy to do so at this time. A repository containing the raw as well as the subject-level data for the two experiments has been made available on Zenodo at the following link: https://doi.org/10.5281/zenodo.7347842. We also provided this link to the raw data in the main text of the manuscript (see lines 83-84).

Comment 2: I make some specific points about power below but I would describe a major limitation of this work that was not mentioned is that it was powered to detect large effects. Reviewer 1 suggests it had "adequate statistical power" yet this would only apply to large effects. This means it had low power

to detect smaller effects, which might be reflective of typical effects sizes in psychological research, and increase the chances of results reflecting Type 2 error in this case. And it would seem smaller effects (e.g. d = .4) would be still taken as theoretically informative as to the effects of sleep had they been found and sufficient power was available to make them statistically significant. This is an issue for both the performance data and the correlation data. This is reflects a general problem of too optimistic effect size estimates as the basis of power analyses, given we know that published effect sizes will likely be over estimates. While the sample size is typical or even higher than many sleep and memory studies in the past, a key issue for the replication crisis has been the use of small samples (e.g. n < 30 per group) in between-subjects designs. In the case of sleep research, while there are difficulties in obtaining large sample sizes continued use of sample small sample sizes will have negative consequences for the credibility of the field (see Cordi & Rasch, 2021) and increasingly replication studies in the field sleep research are showing challenges here. In this particular case, n = 25 per group would lead to any effect size of Cohen's d < .57 as being non-significant (in a between-subjects t-test). For instance, I calculated the key effect in Table C as a Cohen's d = .51, which was not significant (p = .07). Given estimates that the average effect size in psychology might be around d = .4, the design had poor sensitivity to detect effects around this magnitude. However, the language of the discussion was very categorical in wording such as "no evidence for an effect of sleep," and "no performance benefit", which is too strong, particularly given Null Hypothesis Significance Testing framework employed. More formally, it appears the paper accepts the null hypothesis rather than failing to reject it.

I did find the post-hoc analyses to suggest the relatively large (but not significant) effect in the first experiment seemed due to baseline differences reasonably persuasive, although I believe this was not pre-registered. And the consistency of the pattern of results across both studies is suggestive of a null effect. But if true power was low (i.e. there was a real but moderate effect), then two null effects in a row being a result of Type 2 error should be taken seriously as a candidate explanation for these pattern of results. The general point remains that with small sample sizes it is harder to make strong conclusions about the absence of moderate but realistic effects, particularly given the confidence intervals of effect sizes estimate with small sample studies will be include a large range of plausible effect sizes.

PLOS One is a journal that does welcome null results, and I think these results despite the small sample sizes are worth publishing, but in-line with our editorial policy conclusions should be calibrated to what the data can support. Therefore I would like to see a more measured discussion of these findings in a revision which gives greater weight to the uncertainty present, and gives some credence to alternative explanations for these results in the light of effect sizes and statistical power.

<u>Author response:</u> Please note that we grouped above several comments made by the Editor, as they all fall under the general umbrella of statistical power / sample size. [Also, please see our response to

Comment 3 below with respect to the details provided in the sample size justification.] We appreciate the editor's thoughtful comments regarding the sample sizes in this research. We acknowledge that the sample size justification was indeed based on detecting a relatively large effect size. Accordingly, we added additional information on this matter in the limitations section (see lines 614-619 of updated manuscript; and copied below for completeness).

"Finally, while our sample size for the current experiments was computed based on previous work employing the same motor task(King et al. 2019), it is worth explicitly stating that we were powered to detect a relatively large effect (i.e., Cohen's f = 0.383). Accordingly, it is feasible that statistical power was not sufficient to detect smaller effects. Nonetheless, it should be emphasized that the sizes of our experimental groups are larger than the vast majority of sleep and motor memory research (Schmid et al. 2020) and that our results were consistent across two independent experiments. "

To address some of the more specific comments made by the editor; we agree that the results of Experiment 1 revealed a moderate effect in favor of a beneficial effect of sleep (Table 2C; Group main effect, partial η^2 = 0.067; the editor computed as Cohen's d = 0.51). However, and as later noted by the editor, this group difference was largely driven by pre-intervention differences. When these differences were accounted for, the effect size dropped to partial η^2 = 0.028 (see Figure S2), which would be considered closer to a "small" effect size. Note that the analogous result for Experiment 2 (Table 3C) resulted in a partial η^2 < 0.001. In summary, the main contrasts indicated effect sizes that can be considered small so it is unlikely that our null results may be attributed solely to "small" sample sizes.

We do agree with the editor that we should be more careful with the wording used at times in our manuscript, especially given the Null Hypothesis Significance Testing framework employed. We have made changes in the discussion to this effect of updated manuscript (e.g., lines 540-541, 622-624).

Last, we wish to emphasize that the discussion does propose an explanation that is alternative to sleep exerts no effect. Specifically, we suggest that the effects reported here could be due to the timing of the retest in the current study and thus our results could differ if our assessment of integration occurred following a longer delay (see lines 581-595).

Comment 3: The power analysis states the "primary effect of interest in this earlier work was the group difference in performance on the novel transitions learned in the Session 2 training phase, reflecting integration processes."
It would be helpful to elaborate in his section more on the exact statistical method and contrast that represents the primary effect of interest (i.e. an ANOVA, a t-test etc...), including the dependent

measure(s). One issue is "performance" is underspecified, given the results appear to use both RT and errors as performance measures. I believe it may mean RT in this context but it should be stated explicitly if RT was the primary test of your hypothesis. This could also be addressed in the introduction in reference to the previous work - did it find significant effects with RT and with accuracy or just RT? If the latter, then I presume the hypotheses here were focused on the RT effect, and this should be clarified.

"This group difference presented a Cohen's d effect size of 0.86."

On my examination of King et al. (2019) it was not straightforward to identify where this Cohen's d came from partly because the exact test is underspecified (see above point). But it appeared that there was no direct comparison between a 24 period consolidation group in Experiment 1a, and an intermediate group in Experiment

Addressing the above point may help here but it should be made more clear how this value was determined, which made some clarification on how the design of the studies differed. In fact, from my understanding it seems misleading to state the "primary effect of interest in this earlier work was the group difference" if there no was test of the group difference, thought this could result from ambiguity in the wording of this statement. It seems instead that the study involved a significant effect in Experiment 1a, and a non-significant effect in Experiment 2, but without direct comparisons one cannot conclude they are different (e.g. see Gelman & Stern, 2012). This should be highlighted as otherwise the impression could be misleading.

Trying to make sense of this in the present work, it seems like it could refer to the analysis on p. 22 "Last, repeated-measures ANOVAs with the between-subject factor group and the within-subject factor transition type (novel vs. learned) conducted on the Session 2 test revealed no group differences in performance on the two transition types after extensive task practice". Yet this would not be a test of performance just on novel transitions, as it includes novel vs. learned as a factor in an ANOVA, rather than just a between-subjects comparison on RT for novel transitions in Session 2 training. But Table 3 section C does appear to show this contrast referred to in the participants section as the key effect, with the group difference of $F(1, 48) = 3.31$, $p = .07$. However, this does not seem to match what is described in the text. Could this be clarified in the manuscript.

<u>Author response:</u> Similar to above, please note that we grouped here the comments made by the editor that are centered around details of the sample size justification / computation. We acknowledge that our rationale lacked detail and thank the editor for bringing this to our attention. Also, while revisiting our calculations, we discovered that the sample size computation in the original pre-registration used an incorrect outcome variable. Specifically, the computation was based on a speed-accuracy aggregate measure referred to as Performance Index (PI) that was used in earlier versions of

the referent paper(King et al. 2019). The sample size computation should have been based on response time (RT), the primary outcome variable in the current research. We re-computed sample size based on RT; and, importantly, this did not ultimately result in a different sample size. To address the error, we created an addendum to our pre-registration. We followed the procedure outlined by the Open Science Framework (OSF; update registration when the timestamp is important; https://help.osf.io/article/156-update-your-registration). The amendment, as well as the original, is retrievable and transparently logged through OSF at the following link: https://osf.io/ej6ga/registrations.

We have also updated our sample size rationale in the manuscript text (see lines 98-110 in updated manuscript). This is copied below for completeness. Please also note that this updated text provides additional clarifications with respect to the specific contrast and values used in the sample size computation.

"As the specific effects of sleep on the integration of novel motor information into memory has not been previously investigated, a literature-based a priori estimation of the hypothesized effect size is not possible. Nonetheless, given the identical task and an analogous experimental design, sample sizes were determined on the basis of the effect size measured in our previous work investigating the influence of compatible vs. incompatible ordinal structure on motor memory integration (King et al., 2019 Psych Sci). The primary effect of interest in this earlier work was the group main effect in the response time (RT) for novel transitions in the session 2 training phase, as assessed with a 2 (compatible vs. incompatible) x 20 (practice blocks) ANOVA. This group main effect was significant ($F(1,36)=5.29$, $p=0.027$, $\eta p2=0.128$ / Cohen's F = 0.383). Corresponding power analysis (Effect size f=0.383, tails = 2; alpha=0.05, Power=0.80, correlation among repeated measures = 0.72) via the software G*Power (Faul, Erdfelder, Lang and Buchner, 2007) then resulted in an estimated 21 subjects per group. In Experiment 1, we aimed to include 25 subjects per group to be slightly more conservative."

Comment 4: The design of the study sets up a difference in memory integration for the novel sequences, compared with memory retention for the learned sequences. However, the discussion seems exclusively focused on integration (e.g. wording of first paragraph of the discussion). While I understand it might be the primary focus, it may be useful to at least mention for retention as well in the discussion section as it seemed to be important to the design and a worthwhile question on its own, especially if you might expect retention to be a prerequisite for integration.

Author response: We thank the editor for giving us the opportunity to discuss retention processes. We have added a discussion of the related findings on lines 593-600 of the updated manuscript (copied below for completeness).

"In addition to finding no group differences in the integration of new motor information into a compatible schema, we similarly found no effect of sleep on retention of previously learned information

(i.e., performance on the learned transitions). This result is not consistent with extensive literature detailing a beneficial effect of sleep on memory consolidation and retention processes (Rasch and Born 2013). However, it is worth noting that our experimental design is distinct from that generally employed in memory retention studies, as previously learned information is here embedded in a novel context. Accordingly, our design is optimized to test integration of novel information into an existing schema and the impact on the assessment of previously learned motor information is unclear."

Comment 5: "In conclusion, we tested whether sleep is necessary for fast integration of novel motor information into a previously acquired cognitive-motor schema. " I struggled with this sentence as it doesn't appear this is what was tested, and what such a test would entail. What was actually tested was whether sleep lead to better performance compared with a wake group on a range of measures. Instead, a test for if sleep is "necessary " might take the form of some kind of test to show that a group without sleep were not able to show "fast integration". But the results all appeared to show group contrasts. This seems like the framing could be more relevant to the design of the King et al. (i.e. the compatible vs non-compatible contrast) study rather than this study. This should be addressed.

Author response: We thank the editor for pointing out our unclear wording in this sentence. We have removed the wording surrounding "fast" integration and whether sleep is "necessary". The updated sentence now reads as: "We tested whether sleep influences the integration of novel motor information into a previously acquired cognitive-motor schema" (lines 620-621).

Reviewer #1:

Comment 1: The data collection and analyses plan for Experiment 1 was pre-registered, giving more confidence in the tests conducted. Experiment 2 was exploratory but the data analysis followed that of the pre-registered Experiment 1. Furthermore, the sample sizes for both experiments were determined on the basis of the effect size measured in a similar study, ensuring adequate statistical power for each experiment. The experiments are also well controlled, with a habituation night for Experiment 1 and wake conditions for each experiment. Overall, this is an interesting and well-controlled piece of work that addresses an important question in the field.

Author response: We thank the reviewer for acknowledging the positive contributions of our research.

Comment 2: However, my main concern is whether a schema-facilitated motor memory consolidation is possible with the current design as the authors do not control for task interference. The authors state that "Sequence 2 was highly similar to Sequence 1, as 75% of the elements were in the same ordinal position. The positions of keys 2 and 3 (represented in red) were switched in Sequence 2, resulting in 4 novel key transitions to be learned relative to Sequence 1." Is changing the position of 2 keys enough to consolidate the new sequence as a separate memory trace that is compatible with and integrated

into a pre-existing schema? Or would the learning of new sequences result in an updated representation of the original one? I strongly suggest the authors discuss the results in the context of task interference between competing memory traces.

<u>Author response:</u> We thank the reviewer for this interesting comment. We wish to emphasize that the design employed in the current research closely followed that from our previous behavioral study(King et al. 2019). This research demonstrated a performance enhancement when performing this schema-compatible sequence (the same used in our current experiments, with the position of only two keys switched compared with Sequence 1) compared with those performing a schema-incompatible sequence. The original study also included a control group of participants performing the same sequence in both sessions, and found that performance of this same-sequence group was comparable with that of the schema-compatible group. These data are consistent with an interpretation in which Sequence 1 serves as a schematic scaffold upon which Sequence 2 is rapidly integrated (i.e., learning of Sequence 2 updated the representation). It is also worth noting that this research was intentionally designed to mimic – albeit adapted for motor sequence learning in humans – the seminal rodent work by Tse, Morris and colleagues that forms the experimental backbone of the schema model of memory consolidation(Tse et al. 2007, 2011).

The reviewer did explicitly bring up the notion of task interference. We agree that it is feasible to discuss our data in the context of interference between sequences. However, our design employs a specific type of potential interference; namely, a second sequence that shares an underlying structure with the first sequence. Thus, it is our opinion that discussing our results in the context of a schema is more appropriate.

Comment 3: Line 32 & 72: the authors investigate the relationship between NREM sleep duration and post-sleep behavioural performance. However, the results of reference (6) suggest that REM sleep "plays a crucial role in the rapid consolidation of schema-conformant items". Thus, it is unclear why the relationship with REM sleep was not investigated and I suggest the authors test these correlations as well.

<u>Author response:</u> These hypotheses were based on studies demonstrating that NREM sleep, and sleep characteristics that are prevalent in NREM sleep (i.e., spindles and slow waves), are crucial for schema formation and integration processes (Hennies et al. 2016; Tamminen, Lambon Ralph, and Lewis 2013). As Experiment 1 was a nap study and not an overnight study, we did not expect participants to display high amounts of REM sleep. Indeed, only 10 participants reached the REM sleep stage, with an average REM duration of approximately 5 minutes. Consequently, we are extremely underpowered to detect

any relationship between REM sleep and post-sleep performance. For completeness, correlation coefficients and corresponding (uncorrected) p-values are shown below. Given the small number of participants who reached REM sleep and the limited duration of this sleep stage, it is our opinion that these correlations are not suitable to offer any meaningful interpretation and thus we have elected to not add these results to the main text.

	**REM duration (min)**
**Performance Index**	
All transitions	r=-0.07; p=0.75
Learned transitions	r=-0.02; p=0.94
Novel transitions	r=-0.10; p=0.64
**Response time**	
All transitions	r=0.08; p=0.70
Learned transitions	r=0.02; p=0.91
Novel transitions	r=0.12; p=0.56
**Accuracy**	
All transitions	r=0.20; p=0.34
Learned transitions	r=0.12; p=0.56
Novel transitions	r=0.15; p=0.49

Comment 4: Line 161-164: sleep is known to benefit implicitly learned SRTT but only if the learning is contextual (Spencer et al., 2006 , Current Biology). The methodological section of the manuscript suggests that the cues were all squares and had the same colour during practice blocks. Have the authors made sure to include contextual association in their task? The lack of such could have contributed to the lack of group effect and should be discussed.

Author response: There appears to be some confusion with respect to the motor task employed. Participants were explicitly told that there was a fixed, repeating sequence (see Methods – Motor task section on lines 159-174 of the manuscript). Accordingly, our task was more similar to the Explicit condition in the Spencer et al. 2006, which also demonstrated a beneficial effect of sleep.

Comment 5: Line 212-216, 442 and 515: please clarify what was the aim of running the generation task. Furthermore, the generation task employed by the authors does not allow to assess the explicit

memory of the sequence. Rather, the participants were asked to self-generate the sequence using the same keys that they used to perform the SRTT. Thus, the sequence could have been generated implicitly during the generation task, even in the absence of visual cues. To test the explicit memory of a sequence, one would have to eliminate the sequence-related motor system activity, e.g., by asking the participants to mark sequence order on printed out screenshots of the task, as in Cousins et al. (2014). Thus, I would suggest referring to the task as 'generation task' and avoid using terms like 'explicit awareness' (line 515 and 441).

Author response: We thank the reviewer for this comment. Our intention with the generation task was to assess sequence knowledge and we indeed cannot rule out the possibility that both explicit and implicit processes contribute to the observed results. We have altered the wording in the text to convey that this task assesses "sequence knowledge" as opposed to "explicit awareness." Examples of these changes and additions can be found on lines 215-6, 453-4 and 530-1 of the updated manuscript as well as in Tables S3 and S12 in the supplement.

Minor comments:

Comment 6: Please state the sample size for each analysis in the figure/table legend.

Author response: Sample sizes have been added to all figures and tables both in the main text and in the supplementary materials.

Comment 7: Line 71: it would be helpful to know what the authors mean by 'enhanced integration' here – is it faster/more accurate behavioural performance on the second sequence? If so, I suggest clarifying this in brackets.

Author response: Following the reviewer's suggestion, this phrase now reads as: "enhanced integration (i.e., decreased response time)" (line 69 in updated manuscript).

Comment 8: Line 102-104: "The primary effect of interest in this earlier work was the group difference in performance on the novel transitions learned in the Session 2 training phase, reflecting integration processes." – consider rephrasing this sentence as it is hard to grasp.

Author response: This sentence was changed to: "The primary effect of interest in this earlier work was the group main effect in the response time (RT) for novel transitions in the session 2 training phase, as assessed with a 2 (compatible vs. incompatible) x 20 (practice blocks) ANOVA.", found at lines 102-105 of the updated manuscript.

Comment 9: Line 87-120: please be consistent with the use of words vs numerals for numbers.

Author response: Numbers are now indicated by numerals unless used at the beginning of a sentence.

Comment 10: Line 132: if I'm not mistaken, this is the first time when the term "SRTT" is introduced. If so, please explain the abbreviation here and use "SRTT" (instead of "SRT task", e.g., line 372, 380) throughout the manuscript.

Author response: Mentions of "SRT task" throughout the manuscript have been converted to "SRTT", and we have spelled out the acronym at the first mention.

Comment 11: Line 136: replace "design: participants" with "design. Participants" for consistency with line 139.

Author response: We thank the reviewer for catching this inconsistency. It has been corrected.

Comment 12: Line 143: to avoid confusion, please specify in the figure legend that the thumbs were excluded from the numbering system.

Author response: This has been added to the figure caption.

Comment 13: Line 234: why were channels Fz, PZ and Oz omitted during habituation?

Author response: The purpose of the habituation nap was simply to allow participants to familiarize themselves with the sleep laboratory environment, and to check whether they were able to fall asleep under these conditions. The data from this session are not used in data analyses, given known effects of acclimating to PSG recordings in the laboratory environment. Therefore, we opted to use a lighter PSG montage during habituation to speed up the procedure. We have added this information to the manuscript (see lines 236-238).

Comment 14: Line 235: what do the authors mean by "a supplemental individual EEG channel"?

Author response: Channel A1 served as an option to re-reference data if any problems arose with the original reference (Channel A2). This information has been added to the manuscript (see lines 240-241).

Comment 15: Line 384-385: it is a bit confusing to introduce new exclusion criteria in the figure legend. I suggest moving that sentence to the relevant paragraph in the methods section and then just stating the final sample size for each session in the figure legend.

Author response: We have moved this sentence to the participants section (see lines 112-113).

Comment 16: Line 389: consider referring to Table 3A in the brackets as well "(Figure 2, Session 1; Table 3A)".

Author response: The reference to Table 3A has been added. See line 399 of the updated manuscript.

Comment 17: Line 411: it is perhaps worth noting here that the authors also found a significant block effect for accuracy on novel but not learned transitions, perhaps pointing to a decreasing difficulty of the novel transitions over time.

Author response: We thank the reviewer for suggesting this addition, which has now been added to the manuscript text (see lines 421-423 in updated manuscript).

Comment 18: Line 424: the authors might want to make the order of the results consistent with that of the results reported above, i.e., F(1,48)=2.58, p=0.12, n2=0.051 (currently the order of p and n2 is flipped).

Author response: We thank the reviewer for catching this inconsistency. It has now been corrected in the manuscript text (lines 436-437).

Comment 19: Line 428: it is a bit unclear which results this paragraph refers to. Please refer to Table 3 in brackets to avoid such confusion.

Author response: We apologize for the confusion. The reference to Table 3 has been added to the manuscript text (line 438).

Comment 20: Table S8, table legend: the authors state that "No significant Group, Session or Group by Session effects were revealed", whereas in fact the effect of session was revealed for PVT. The authors note this in the next sentence but I believe the former one should be edited to reflect this as well. Furthermore, the authors say "Note that the significant effect of Session for the measure PVT was driven by overall decreased response time in Session 2 (276 ± 44 ms, compared with 286 ± 55 ms in Session 1), likely reflecting increased familiarity with the task." – I am not convinced that a decrease in PVT can reflect increased familiarity with the task. If that was indeed the case, then the PVT results should follow the same pattern in Experiment 1, which they do not. Consider rephrasing or adding appropriate references which would back up such claims.

Author response: Note that this information is now in S10 Table of the updated submission. We thank the reviewer for highlighting the lack of clarity with respect to the Session effect in the PVT. We have updated this in the caption.

The argument about familiarization with the task was intended to convey that RTs could be higher in Session 1, particularly in the beginning, due to first exposure to such a task (i.e., in Session 2, participants are acclimated and thus RTs are faster). We agree with the reviewer that we cannot be certain that this is a correct explanation, so we have simply removed it. This portion of the updated caption now reads as:

"No significant Group, Session or Group by Session effects were revealed with the exception of a significant effect of Session for the PVT. This was driven by overall decreased response time in Session 2 (276 ± 44 ms, compared with 286 ± 55 ms in Session 1). Importantly, this decrease across sessions did not differ between groups, as indicated by the lack of a Session x Group interaction."

Comment 21: Line 470: please refer to Table 4A in the brackets as well.

Author response: The reference to Table 4A has been added to the manuscript text (line 483).

Comment 22: Line 474: the authors state that "Accuracy remained stable and high during both training and test runs, with no differences across groups". However, according to Table 4, block had a significant effect on accuracy at Session 1 Test. Please acknowledge that in text.

Author response: The sentence "There was a significant effect of block on accuracy at Session 1 test (all transitions), caused by decreased accuracy on the last test block (average 89%, compared with 91% in the first three blocks)" has now been moved from the table caption to the main text (lines 487-490).

Comment 23: Line 497: consider replacing 'Table 4' with 'Table 4B-C'

Author response: The reference to Table 4 has been updated to Table 4B-C (line 512).

Comment 24: Line 551-553: As stated by the authors, previous studies have illustrated the involvement of hippocampus in motor sequence learning. However, the SRTT is not purely procedural and includes a declarative component as well (see Robertson 2007, J Neurosci). Thus, I am unsure whether stating that "neuroimaging studies have illustrated the involvement of the hippocampal formation in motor sequence learning" supports the argument that "consolidation processes of declarative and motor memories share common features and rely on similar neural substrates and networks" – the SRTT is just a combination of both, isn't it?

Author response: We thank the reviewer for raising this interesting point. We do not disagree with the reviewer; namely, that there may be a "declarative" component of motor sequence learning. However, the SRTT is still considered a **motor** task, and many in the field still consider learning and memory processes in the motor domain to be distinct (anatomically and functionally) from the declarative domain. The reviewer's primary point is in line with our assertion; the traditional dichotomization that is still present in today's literature is incomplete at best (and inaccurate at worst). This is indeed what

we aimed to highlight, that consolidation following motor and declarative tasks are not distinct. We agree with the reviewer that we could have been clearer with the phrasing in the manuscript text. We have changed the sentence "the consolidation processes of declarative and motor memories share common features and rely on similar neural substrates and networks" to "the consolidation of memories formed during motor tasks share common features and rely on similar neural substrates and networks as the consolidation of memories formed during declarative tasks." (lines 566-568). This effectively shifts the emphasis to the tasks employed as opposed to the mnemonic processing.

Comment 25: Line 525: please replace 'in motor performance' with 'in post-sleep motor performance' or similar.

Author response: The phrase "in motor performance" has been updated to "in post-sleep motor performance" (line 540).

Reviewer #2:

Comment 1: This is a very rigorous and well-conducted experiment. The only issue I noticed is that the link to the raw data currently requires permission to access, so it is not publicly available.

Author response: We thank reviewer #2 for the positive assessment of our article. A repository containing all single-subject data for the two experiments has been made available on Zenodo at the following link: https://doi.org/10.5281/zenodo.7347842.

Reviewer #3:

Comment 1: In this study Reverberi and colleagues tested in two experiments whether diurnal or nocturnal sleep after the acquisition of a motor schema would facilitate the integration of new, schema-congruent motor information. Interestingly, (when compared to wakefulness) sleep did not exert any beneficial effect on the integration of novel motor information. In addition, there was no significant relationship between NREM sleep features and memory performance. This is an interesting and timely study. The manuscript is well written and the applied methods are sound.

Author response: We thank the reviewer for these positive comments.

Comment 2: The authors state that the computed densities and amplitudes of spindles and slow oscillations were averaged across channels Fz, Cz, and Pz. What is the rationale of this procedure? Why not just using Fz for SOs (were they are dominant) and Cz for spindles?

Author response: We thank the reviewer for raising this point. We agree with the reviewer that there could be a number of potential options with respect to the selection of EEG channels. The option proposed by the reviewer is certainly appropriate. We opted to use an average of the midline channels to be consistent with our previous research(Nicolas et al. 2022) and to limit the number of tests employed (i.e., not assessing multiple channels independently). We certainly agree with the reviewer that providing single-channel data could be of interest to the readers. We have added exploratory correlation analyses performed on single-channel Fz, Cz, and Pz data in the Supplementary Tables S7-S9, respectively. Consistent with our original approach, we applied False Discovery Rate (FDR) correction within each family of tests. These FDR-corrected p-values are provided in Tables S6-S9. No correlation survived correction for multiple comparisons. [It is worth noting that the correlation between spindle amplitude and the post-sleep performance index for novel transitions was negative and marginally significant for channel Cz (r=-0.49; *p*=0.052).] Single-channel results are now referenced in the main text of the manuscript in lines 467-469.

We would like to also note here that re-examining our sleep data revealed some data averaging errors which resulted in incorrect values being initially reported in Tables 2 and S6. The values have now been corrected. Importantly, the changes did not alter the conclusions drawn from the reported results and thus the primary conclusions offered in the original version are still applicable.

Comment 3: The authors state that all participants reached sleep stage N2. How many of the participants reached SWS and REM? If the authors would split their sample into participants reaching (for example) SWS and those who did not, would memory performance differ between the groups?

Author response: We thank the reviewer for pointing out that this information was missing. It has now been added to the caption of Table 2: "N=25 for all measures except for duration of NREM3 and REM. Only 13 and 10 participants reached NREM3 and REM sleep, respectively."

Given that we did not have specific hypotheses regarding sleep stages N3 and REM and the small amount of time each participant spent in these stages (averages of approximately 8 minutes in N3 and 5 minutes in REM), it is our opinion that splitting the data based on these metrics is difficult to justify. For completeness, however, comparison graphs showing post-sleep behaviour of participants reaching stage N3 versus all other Nap group participants, as well as those reaching stage REM versus all other Nap group participants, are shown in the figures below. There was no significant difference in response time between these groups (N3 vs no N3: main effect *group*: $F(1,23)=0.003$, $p=0.96$, $\eta^2=0.000$; *group* x *block* interaction: $F(4.82,110.92)=1.09$, $p=0.37$, $\eta^2=0.045$; REM vs no REM: main effect *group*: $F(1,23)=0.03$, $p=0.87$, $\eta^2=0.001$; *group* x *block* interaction: $F(4.78,109.92)=0.58$, $p=0.71$, $\eta^2=0.025$). For the reasons outlined above (i.e., lack of specific hypotheses, limited number of participants reaching

such stages and limited durations of these sleep stages), we have elected to not include these comparisons in the updated manuscript.

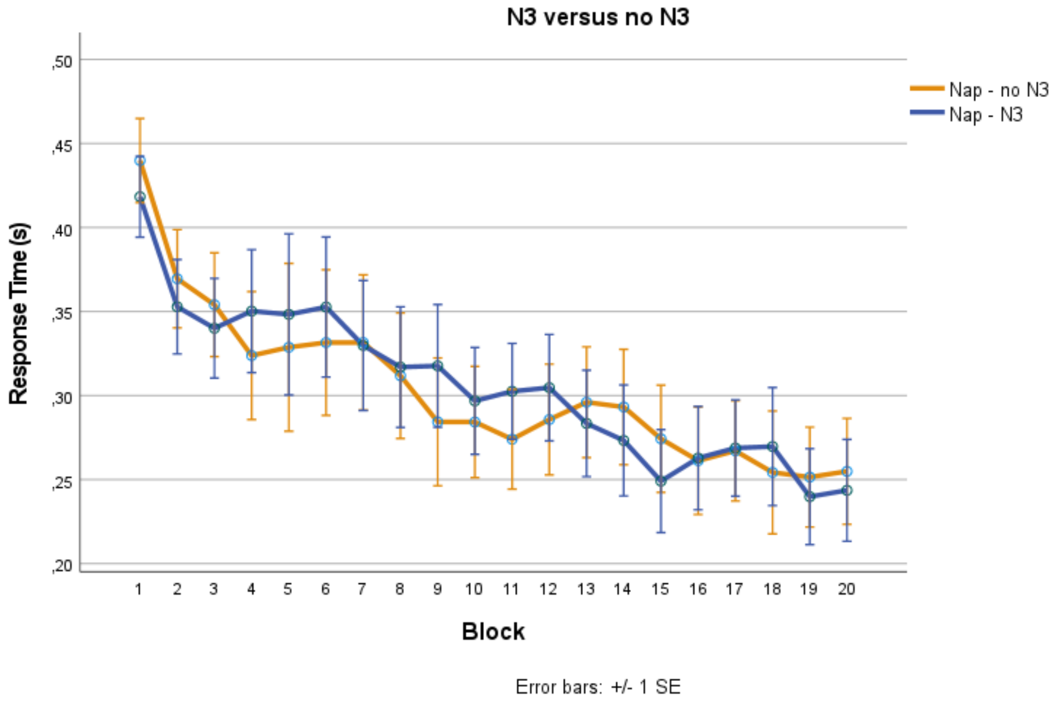

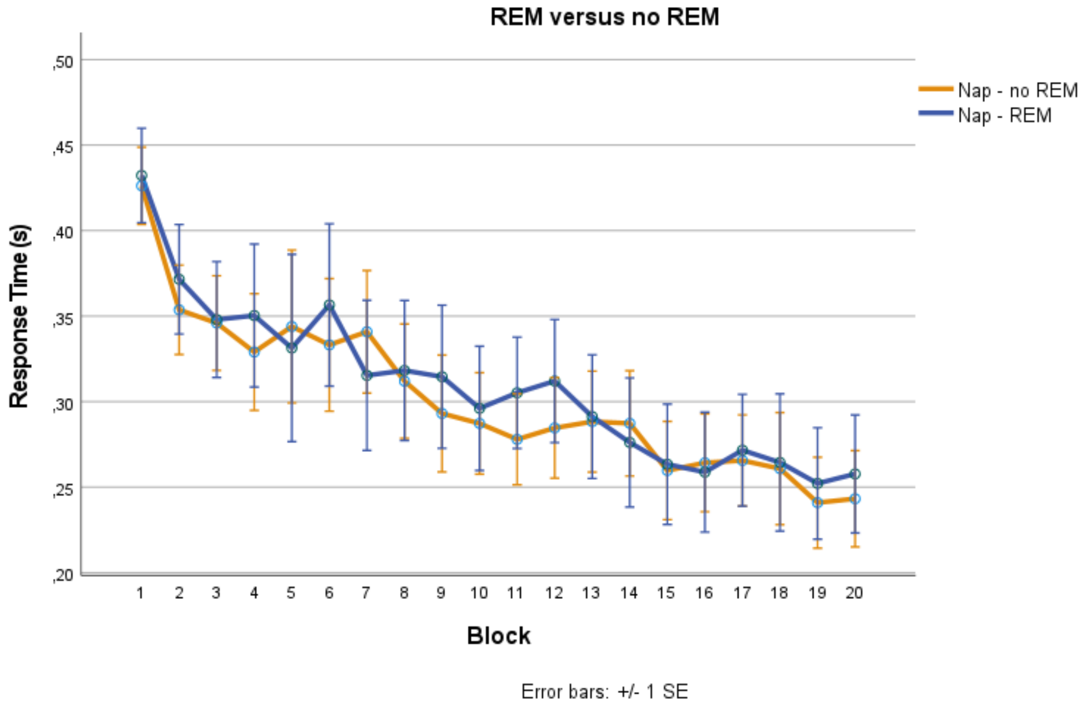

References:

Hennies, Nora, Matthew A. Lambo. Ralph, Marleen Kempkes, James N. Cousins, and Penelope A. Lewis. 2016. "Sleep Spindle Density Predicts the Effect of Prior Knowledge on Memory Consolidation." *Journal of Neuroscience* 36(13):3799–3810.

King, Bradley R., Nina Dolfen, Mareike A. Gann, Zenzi Renard, Stephan P. Swinnen, and Genevieve Albouy. 2019. "Schema and Motor-Memory Consolidation." *Psychological Science* 30(7):963–78.

Nicolas, Judith, Bradley R. King, David Levesque, Latifa Lazzouni, Emily Coffey, Stephan Swinnen, Julien Doyon, Julie Carrier, and Genevieve Albouy. 2022. "Sigma Oscillations Protect or Reinstate Motor Memory Depending on Their Temporal Coordination with Slow Waves." *ELife* 11.

Rasch, Björn and Jan Born. 2013. "About Sleep's Role in Memory." *Physiological Reviews* 93(2):681–766.

Schmid, Daniel, Daniel Erlacher, André Klostermann, Ralf Kredel, and Ernst Joachim Hossner. 2020. "Sleep-Dependent Motor Memory Consolidation in Healthy Adults: A Meta-Analysis." *Neuroscience & Biobehavioral Reviews* 118:270–81.

Tamminen, Jakke, Matthew A. Lambon Ralph, and Penelope A. Lewis. 2013. "The Role of Sleep Spindles and Slow-Wave Activity in Integrating New Information in Semantic Memory." *Journal of Neuroscience* 33(39):15376–81.

Tse, Dorothy, Rosamund F. Langston, Masaki Kakeyama, Ingrid Bethus, Patrick A. Spooner, Emma R. Wood, Menno P. Witter, and Richard G. M. Morris. 2007. "Schemas and Memory Consolidation." *Science* 316(5821):76–82.

Tse, Dorothy, Tomonori Takeuchi, Masaki Kakeyama, Yasushi Kajii, Hiroyuki Okuno, Chiharu Tohyama, Haruhiko Bito, and Richard G. M. Morris. 2011. "Schema-Dependent Gene Activation and Memory Encoding in Neocortex." *Science* 333(6044):891–95.
